# Comparative efficacy of different types of antihypertensive drugs in reversing left ventricular hypertrophy as determined with echocardiography in hypertensive patients: A protocol for systematic review and meta-analysis

**DOI:** 10.1097/MD.0000000000026455

**Published:** 2021-06-25

**Authors:** Hao Xu, Bo Hu, Wulong Wu, Yong Jin

**Affiliations:** a Department of Ultrasound; b Department of Endocrinology, The People's Hospital of Fenghua; c Department of Ultrasound, Ningbo No. 1 Hospital, Ningbo, Zhejiang, China.

**Keywords:** antihypertensive drugs, echocardiography, left ventricular hypertrophy, meta-analysis

## Abstract

**Background:**

Reversing left ventricular hypertrophy (LVH) can reduce the incidence of adverse cardiovascular events. The lack of direct comparison between different antihypertensive drugs cannot evaluate the superiority-inferiority differentiation of different antihypertensive drugs in reversing LVH. Therefore, the objective of this protocol for systematic review and meta-analysis was to compare the efficacy of different types of antihypertensive drugs in reversing LVH in hypertensive patients.

**Methods:**

This meta-analysis was conducted in accordance with the guidelines of the Preferred Reporting Items for Systematic Reviews and Meta-Analyses Protocols statement guidelines. Studies were identified through systematic searches in June 2021 with no restrictions on date and time, language, and publication status using the following bibliographic databases: Embase, Medline, PubMed, Web of Science, Science Direct, and the Cochrane Library. The risk of bias assessment of the included studies was performed by two authors independently using the tool recommended in the Cochrane Handbook for Systematic Reviews of Interventions (version 5.1.0). All calculations were carried out with Stata 11.0 (The Cochrane Collaboration, Oxford, United Kingdom).

**Results:**

The results of this systematic review and meta-analysis will be published in a peer-reviewed journal.

**Conclusion:**

We hypothesized that the use of angiotensin-converting enzyme inhibitors or angiotensin receptor blockers in antihypertensive therapy could achieve better efficacy in reversing LVH in hypertensive patients.

## 1 Introduction

Hypertension is a major risk factor for cardiovascular disease and left ventricular hypertrophy (LVH) detected by echocardiography is a common target organ damage of hypertension, which can cause abnormal changes in the ultrastructure and energy metabolism of cardiomyocytes, resulting in adverse cardiovascular events such as abnormal cardiac contraction and diastolic function, and arrhythmia.^[1–3]^ A definite association has been established, not only between hypertension and LVH, but also between LVH and cardiovascular events, both of which, cumulatively and independently, increase the risk of cardiovascular mortality and morbidity.^[4]^ Pharmacotherapy directed at reducing elevated blood pressure can reduce complications associated with hypertension. It has also been observed that with prolonged control of blood pressure, there is regression of LVH with significant decrease of unfavorable clinical outcomes.^[5,6]^ On the basis of preliminary clinical studies, the American expert consensus on hypertension points out that angiotensin receptor blockers (ARB) or angiotensin-converting enzyme inhibitors (ACEI) are generally used in hypertensive patients with LVH.^[7]^

Several clinical studies have shown that there has been controversy over whether patients with hypertension can reverse LVH and the pros and cons of reversing LVH after treatment with antihypertensive drugs.^[8,9]^ This also brings great confusion to clinical decision makers in the treatment of hypertensive LVH which antihypertensive drugs can obtain the maximum benefit. Present conclusion should be considered with caution as many studies included in these analyses were noncomparative or nonrandomized, and of too small a size. At the same time, the lack of direct comparison between different antihypertensive drugs cannot evaluate the superiority-inferiority differentiation of different antihypertensive drugs in reversing LVH. Therefore, the objective of this protocol for systematic review and meta-analysis was to compare the efficacy of different types of antihypertensive drugs in reversing LVH in hypertensive patients.

## 2 Methods

This meta-analysis was registered at Open Science Framework registries (registration number: 10.17605/OSF.IO/Q6P2R) and was conducted in accordance with the guidelines of the Preferred Reporting Items for Systematic Reviews and Meta-Analyses Protocols statement guidelines.^[10]^ Ethics application was not required as this study is based on published trials.

### 2.1 Search strategy

Studies were identified through systematic searches in June 2021 with no restrictions on date and time, language, and publication status using the following bibliographic databases: Embase, Medline, PubMed, Web of Science, Science Direct, and the Cochrane Library. The following search terms were used: hypertension; LVH; and each class of antihypertensive drugs. The reference lists of the included studies were also checked for additional studies that were not identified with the database search.

### 2.2 Inclusion and exclusion criteria

Two independent researchers removed duplicated articles by using EndNote and then screened the titles and abstracts of articles to exclude irrelevant studies. Then they reviewed the full-texts of the remaining records independently to determine eligibility for this meta-analysis according to following inclusion criteria: comparisons of 6 classes of antihypertensive drugs were performed and did not include any other non-drug treatment modality; the shortest follow-up time was 3 months; randomized controlled studies; and LVMI was evaluated by echocardiography. Any one of the following articles can be excluded: observational studies, animal experiments, case reports, reviews and other nonrandomized controlled trials; the full text cannot be obtained; and a lack of complete data;

### 2.3 Data extraction

Two investigators reviewed all the titles and abstracts independently. Data was extracted from eligible full-text studies. The data included study population, demographical characteristics, year of publication, country, age, sex, intervention regimens, duration of follow-up, and study outcomes. The main outcomes were left ventricular mass index, regression of left ventricular pressure, and adverse events. If the data are missing or cannot be extracted directly, we will contact the corresponding authors to ensure that the information integrated.

### 2.4 Quality evaluation

The risk of bias assessment of the included studies was performed by two authors independently using the tool recommended in the Cochrane Handbook for Systematic Reviews of Interventions (version 5.1.0).^[11]^ This tool included seven aspects which were sequence generation (selection bias), allocation sequence concealment (selection bias), blinding of participants and personnel (performance bias), blinding of outcome assessment (detection bias), incomplete outcome data (attrition bias), selective outcome reporting (reporting bias), and other bias (baseline balance and fund). Additionally, each of the aspects was ranked low risk of bias, high risk of bias, and unclear risk of bias.

The evidence grade was assessed using the guidelines of the GRADE (Grading of Recommendations, Assessment, Development, and Evaluation) working group including the following items: risk of bias, inconsistency, indirectness, imprecision and publication bias. GRADE pro Version 3.6 software is used for the evidence synthesis.

### 2.5 Statistical analysis

The risk differences with 95% confidence intervals were calculated for dichotomous data, and the weighted mean difference with 95% confidence interval was calculated for the continuous data. Heterogeneity between the studies was assessed by the *χ*^2^ test (significant level of *P* < .10) and the *I*^2^ statistic (*I*^2^ > 50% indicating significant heterogeneity). The results were pooled using the fixed-effect model for *P* > .10 and *I*^2^ < 50% or the random-effect model for *P* < .10 and *I*^2^ > 50%. If significant heterogeneity is found, we will try to explore the source of heterogeneity by subgroup analysis. Publication bias was assessed by drawing contour-enhanced funnel plots. When these plots were not obviously asymmetric, we considered that publication bias was absent. All calculations were carried out with Stata 11.0 (The Cochrane Collaboration, Oxford, United Kingdom).

## 3 Discussion

Prevention or reversal of LVH has been shown to reduce the risk of cardiovascular events in hypertensive patients.^[12,13]^ Several clinical trials and meta-analyses have compared the effects of different classes of antihypertensive drugs on ventricular hypertrophy^[14,15]^; however, the usefulness of the results is limited by their inadequate design and inappropriate methods. Although meta-analyses can improve the statistical power and provide more accurate estimates of the effect value, the results depend largely on the criteria for inclusion in the study. Molecular biology research has shown that LVH in hypertensive patients is a process evolving from quantitative change to qualitative change. This process includes gene translocation of myosin heavy chain, encoding myosin, membrane protein, and energy metabolism of protein gene shift.

ACEI and ARB were reported to be effective in achieving better efficacy in reversing LVH in hypertensive patients. This meta-analysis provides new clues to support the hypothesis that patients with hypertensive cardiac hypertrophy may obtain better clinical benefits from the use of ACEI and ARB as compared with other types of antihypertensive drugs. To improve the quality of life and long-term prognosis of patients with hypertensive cardiac hypertrophy, it is recommended that clinicians choose the optimal antihypertensive drugs to reverse LVH.

## Author contributions

Hao Xu finished the protocol; Bo Hu collect data; Wulong Wu performed data analysis; Yong Jin designed the protocol. All authors approve the final version of the study.

**Conceptualization:** Bo Hu.

**Formal analysis:** Wulong Wu.

**Funding acquisition:** Yong Jin.

**Investigation:** Wulong Wu.

**Writing – original draft:** Hao Xu.

**Writing – review & editing:** Yong Jin.
